# Generation of Monoclonal Autoantibodies From Myelin Oligodendrocyte Glycoprotein-Specific Human B Cells Using an Optofluidic-Based Platform

**DOI:** 10.1212/NXI.0000000000200577

**Published:** 2026-04-29

**Authors:** Anjali J. Panicker, Samuel S. Hinman, Julie Perreau, Ava Aslanpour, Robyn L. Williams, Stephan Steinke, Elizabeth Quinn, Amanda L. Hernandez, Soumya S. Yandamuri, Sarosh R. Irani, Erin E. Longbrake, Kevin C. O'Connor

**Affiliations:** 1 Departments of Neurology and Immunobiology, Yale School of Medicine, Yale University, New Haven, CT;; 2 Bruker Cellular Analysis, Emeryville, CA;; 3 Departments of Neurology and Neurosciences, Mayo Clinic, Jacksonville, FL;; 4 Department of Neurology, Yale School of Medicine, Yale University, New Haven, CT;; 5 Department of Neurology, University of Connecticut Health Center, Farmington, CT; and; 6 Oxford Autoimmune Neurology Group, Nuffield Department of Clinical Neurosciences, University of Oxford, United Kingdom.

## Abstract

**Objectives:**

Monoclonal antibodies (mAbs) are powerful tools for elucidating disease mechanisms. Capturing heterogeneity of patient responses—including clonality, somatic mutations, isotypes, and pathogenic potential—requires building large libraries of mAbs using high-throughput approaches. However, current techniques for identifying and isolating patient-derived mAbs targeting conformational epitopes on membrane proteins remain labor-intensive and inefficient. To address this challenge, we evaluated a cell-based optofluidic antibody discovery pipeline to generate patient-derived mAbs against myelin oligodendrocyte glycoprotein (MOG), a transmembrane autoantigen targeted in MOG antibody-associated disease (MOGAD).

**Methods:**

An optofluidic-based workflow incorporated mammalian display of human MOG (hMOG) in a live cell-based assay (CBA) format. B-cell receptor sequences from individual hMOG-binding B cells were cloned and expressed to generate mAbs from 3 patients. The hMOG binding specificity of these mAbs was validated in off-platform hMOG-CBAs.

**Results:**

hMOG-specific antibody-secreting cells in one patient represented 0.02% of all single B cells screened. From these low-frequency populations, one patient-derived IgG mAb was successfully generated and validated. This IgG mAb, characterized by a high frequency of V-region somatic mutations (5%–12.2%), bound hMOG at concentrations as low as 1 ng/mL.

**Discussion:**

This workflow enables rapid discovery of rare, patient-derived mAbs targeting conformational epitopes on membrane antigens, offering a scalable approach for dissecting autoantibody repertoires.

## Introduction

Autoantibodies are essential diagnostic markers in autoimmunity. Their discovery has transformed neuroimmunology by redefining disease classification, elucidating immunopathologic mechanisms, and informing targeted therapies.^1^ Patient-derived monoclonal antibodies (mAbs) provide molecular-level resolution of disease mechanisms by defining epitope specificity and functional properties that cannot be inferred from polyclonal serum alone, as shown in myasthenia gravis.^2,3^ Similarly, mAbs targeting aquaporin-4 (AQP4)^4^ clarified pathogenic mechanisms in neuromyelitis optica spectrum disorder, while N-methyl-d-aspartate receptor (NMDAR),^5^ contactin-associated protein-like 2 (CASPR2),^6^ and leucine-rich glioma-inactivated 1 (LGI1)^7^ mAbs provided critical mechanistic insights into autoimmune encephalitis.

Myelin oligodendrocyte glycoprotein (MOG) antibody–associated disease (MOGAD)^8^ is a CNS demyelinating disorder characterized by autoantibodies targeting MOG, a transmembrane protein expressed on oligodendrocytes and myelin sheaths.^9^ Accurate detection of MOG-specific autoantibodies requires presentation of native, membrane-bound antigen; consequently, cell-based assays (CBAs) are the clinical gold standard, whereas ELISA or immunoblotting may fail to detect clinically relevant autoantibodies.

Despite their diagnostic and pathogenic relevance, isolating MOG-specific B cells remains technically challenging because of their exceptionally low frequency (∼1 per 10^4^–10^5^ B cells)^10^ and the structural complexity of membrane antigens. Existing approaches for rare B-cell discovery—including soluble fluorescent antigen labeling,^11^ B-cell membrane extraction,^12^ membrane antigen capture,^2^ and single B-cell cloning^13^—are effective but labor-intensive and low-throughput.

To overcome these challenges, we evaluated an optofluidic platform to isolate rare, patient-derived B cells and validate their MOG specificity. This workflow integrates nanoliter-scale wells (“nanopens”) for single B-cell isolation, real-time functional screening using live hMOG CBAs, and B-cell receptor (BCR) sequencing, enabling efficient generation of mAbs against conformationally preserved, membrane-bound antigens. Using MOG—a clinically relevant, structurally complex antigen with few well-characterized human mAbs^14^—as a challenging proof-of-concept target, this platform provides a scalable strategy to isolate rare autoantibodies in MOGAD and other neuroimmunologic disorders.

## Methods

### Human Samples

Cryopreserved peripheral blood mononuclear cells (PBMCs) from 3 MOGAD patients with confirmed MOG autoantibody serostatus and clinical features were used (eTable 1).

### Memory B Cell Isolation and Culture

PBMCs were thawed, and memory B cells were enriched through magnetic-activated cell sorting. Cells were resuspended in activation medium and seeded in 96-well plates. Off-chip activation into antibody-secreting cells was performed over 5 days.

### Optofluidic Single B-Cell Screen, Cell-Based Assay, BCR Sequencing, and Candidate Selection

Antibody-secreting cells (ASCs) were loaded onto OptoSelect 20k chips and isolated into nanopens using optoelectronic positioning. Cells from MOG01 and MOG03 were each loaded onto separate chips, while MOG02 was split across 2 chips. Single ASCs were sequentially assayed for IgG secretion (through capture beads and fluorescence bloom detection) and MOG specificity using human MOG-GFP plasmid-transfected reporter HEK293T cells. Positive cells were exported, lysed, and stored at −80°C for sequencing (eTable 2). First-strand cDNA was generated and amplified. BCRs were amplified with human-specific primers, pooled into libraries, and sequenced on a NextSeq 2000. Candidates with paired heavy and light chains were selected based on quantitative bloom analysis (eTable 3).

### Immunoglobulin Sequence Analysis and mAb Expression

Immunoglobulin heavy and light chain sequence analysis (eTable 4) and mAb expression were performed as previously described.^13^

### Validation of MOG-Specificity Using a Live Human MOG Cell-Based Assay (hMOG-CBA)

mAb supernatants were assessed for MOG binding using 2 complementary approaches. In a flow-based assay, mAbs were incubated with MOG-GFP–expressing HEK293T cells, washed, and stained with a secondary antibody. In a parallel image-based assay, mAbs were incubated with live HEK cells cotransfected with MOG and eGFP, fixed, stained with anti-human IgG and a secondary antibody, and visualized by fluorescence microscopy.

### Standard Protocol Approvals and Patient Consents

Samples were collected, processed, and biobanked at Yale with patient consent and IRB approval.

### Data Availability

Anonymized data published in this article will be made available on request from any qualified investigator.

(Detailed experimental methods are provided in the eMethods.)

## Results

### Optofluidic Screen Identified Patient-Derived mAbs

**Figure 1:**
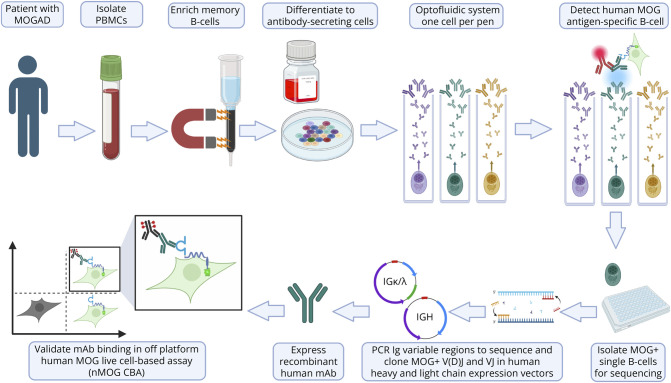
Workflow for High-Throughput Generation and Characterization of MOG-Specific Monoclonal Antibodies From Patients With MOGAD Peripheral blood mononuclear cells (PBMCs) are isolated from patients with MOGAD, and memory B cells are enriched through magnetic separation. These cells are then differentiated into antibody-secreting plasma cells in vitro. Using the Beacon optofluidic system, single cells are captured in nanopens and screened for MOG specificity by testing the binding of secreted antibodies in real-time against live hMOG-GFP-expressing cells. MOG-specific B cells are isolated, and their immunoglobulin (Ig) variable regions (V(D)J for heavy chain and VJ for light chain) are amplified by RT-PCR and cloned into human Ig expression vectors. Recombinant human monoclonal antibodies (mAbs) are expressed and validated off-platform for antigen specificity using a live human MOG cell-based assay (hMOG-CBA).

**Figure 2:**
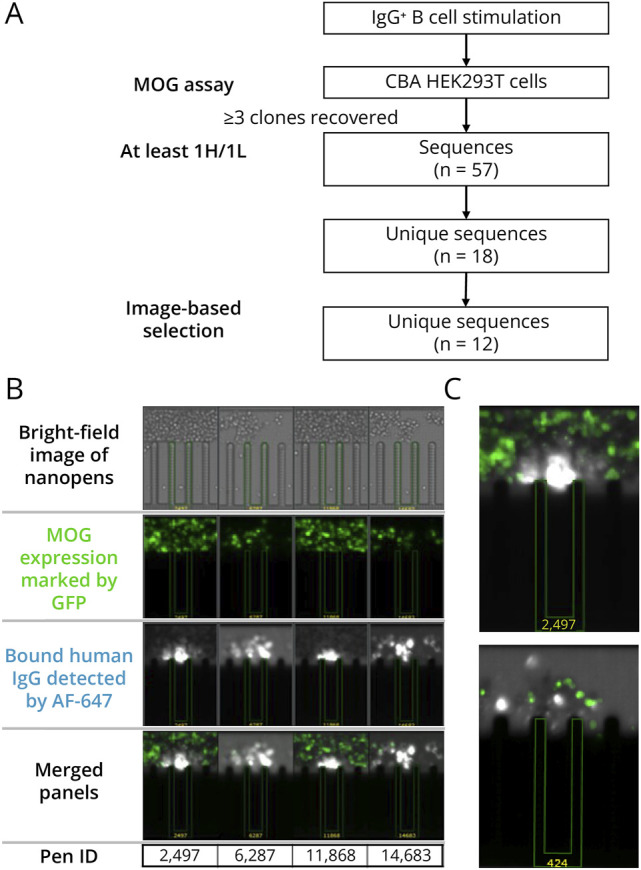
Optofluidic Screening on the Beacon Platform Identifies MOG-Specific Antibody-Secreting B Cells (A) Schematic of the selection process for the final 12 mAb candidates advanced to validation. (B) Individual nanopens containing single antibody-secreting B cells were imaged under bright field (top row). Cells expressing membrane-bound MOG were coincubated and visualized by GFP fluorescence (middle row), while secreted MOG-binding human IgG antibodies were detected using an AlexaFluor-647–labeled secondary antibody (bottom row). PenIDs 2497, 6287, 11868, and 14683 (sharing the identical VH CDR3 sequence) show strong colocalization of GFP+ MOG-expressing cells with bound AlexaFluor-647 signal, indicating MOG-specific antibody secretion. (C) Representative overlapping GFP and AlexaFluor-647 fluorescence images are shown for PenIDs 2497 (positive) and 424 (representative negative well). MOG = myelin oligodendrocyte glycoprotein.

Using MOGAD—a neurologic autoimmune disease where rare B cells produce autoantibodies against the membrane antigen MOG—as a model, we established an optofluidic high-throughput workflow for autoantibody discovery (Figure 1). Memory B cells expanded from the PBMCs of 3 patients were penned as single cells. Autoantibodies binding native membrane MOG were selected using a live human MOG cell-based assay (hMOG-CBA) on the Beacon platform. Fifty-seven paired sequences with ≥3 clones and heavy/light chains were identified; 18 unique sequences remained after accounting for in vitro expanded duplicates (eTable 3). Based on qualitative bloom image data, 12 candidates were selected for validation (Figure 2A). Representative blooms showing GFP+ MOG-expressing cells colocalizing with IgG are shown for mAb 2,497 and related nanopens with identical VH CDR3 (Figure 2B), along with example hMOG-CBA blooms for mAbs 2,497 and 424 (Figure 2C). The frequency of MOG-specific IgG hits was 0.02% (3/14,931 single cells) for MOG01; none for MOG02 or MOG03 (eFigure 1).

### Off-Platform Validation Confirmed mAb 2497 Binding to Clinically Relevant hMOG

**Figure 3:**
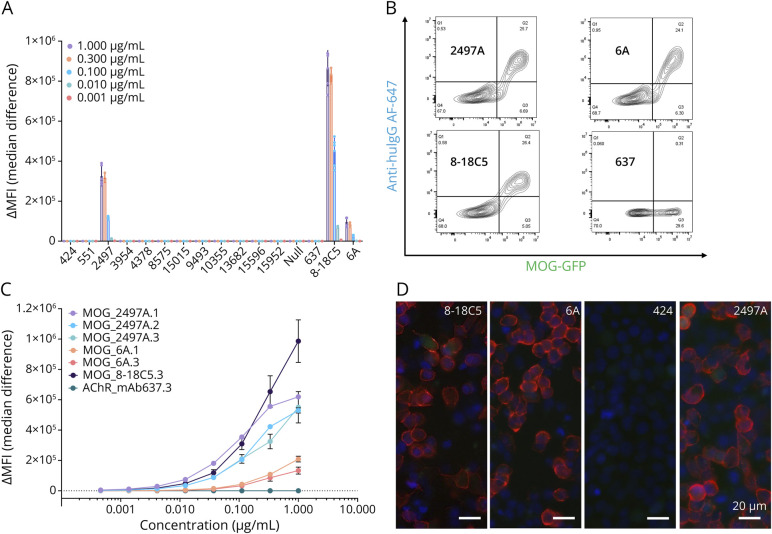
Validation of Candidate mAb 2497 on Human MOG Live Cell-Based Assays (A) Screening of 12 candidate mAbs for MOG binding. HEK293T cells transiently expressing full-length human MOG (GFP+) were incubated with recombinant antibody-containing supernatants collected from 2 independent expression sets, including positive controls (MOG-specific mAbs 8-18C5 and 6A) and negative control (AChR-specific mAb 637). Supernatants were tested at 5 dilutions (1, 0.3, 0.1, 0.01, and 0.001 µg/mL). Antibody binding was detected using AlexaFluor-647–conjugated anti-human IgG and analyzed by flow cytometry. ΔMFI was calculated as the difference in AlexaFluor-647 (AF647) signal between MOG-GFP+ and MOG-GFP− cells. mAb 2497A showed consistent, dose-dependent binding to MOG+ cells across independent expression replicates, confirming specificity. (B) Representative flow plots showing AF647 fluorescence (y-axis) vs GFP fluorescence (x-axis) for mAb 2497A and relevant controls. (C) Binding curves of mAbs on MOG CBA demonstrate reproducible, concentration-dependent MOG binding by mAb 2497A. For external validation, 2 commercial vendors independently manufactured mAb 2497A using only the provided sequence. (Suffix key: 1 = commercial vendor 1, 2 = commercial vendor 2, 3 = in-house production.) (D) Collaborating site (Mayo Clinic) performed independent validation using an image-based hMOG-CBA and confirmed specific binding of mAb 2497A and positive controls (8-18C5 and 6A), while a negative candidate mAb (PenID 424) showed no binding. (Staining key for panel D: blue = DAPI [nucleus], red = AlexaFluor-568 [IgG], green = eGFP [cotransfection marker].) MOG = myelin oligodendrocyte glycoprotein.

The 12 candidate mAbs were expressed and tested in a flow cytometry-based hMOG CBA (Figure 3, A and B). Positive controls (MOG-binding mAbs 8-18C5 and 6A)^14^ and negative control (acetylcholine receptor [AChR]–binding mAb 637) were included. Candidate mAb 2,497 (mAb 2497A, from MOG01) bound hMOG at concentrations as low as 1 ng/mL. Sequence analysis revealed V-region mutation frequencies of heavy (5/99, 5%) and lambda light chains (12/98, 12.2%) (eTable 4). PCR-based subclass analysis identified mAb 2497A as an IgG_1_ (eTable 5). Specificity was confirmed by independent commercial mAb expression from the provided sequences. Purified mAbs confirmed strong hMOG binding (Figure 3C) with no reactivity to AChR, a negative control membrane antigen (eFigure 2). The expressed mAb and controls were deidentified and subjected to blinded analysis at a collaborating site (Mayo Clinic) using an image-based hMOG CBA, confirming robust binding of mAb 2497A and positive controls (8–18C5, 6A),^14^ with no binding for nonreactive mAb 424 (Figure 3D).

### Patient Serum Recapitulates the Binding Characteristics of mAb 2497A

Using established MOG isoforms and point mutants,^14^ we found that the binding profile of mAb 2497A was concordant with the corresponding patient serum (eFigure 3).

## Discussion

This proof-of-principle study demonstrates an optofluidic workflow for rapid discovery of patient-derived mAbs against conformational membrane antigens, exemplified by MOGAD. Streamlining single B-cell functional screening with BCR sequencing, we identified the rare, hypermutated, high-affinity mAb, 2497A, within 2 weeks—substantially faster than months-long conventional approaches. Critically, using live CBAs preserved native membrane conformation, enabling the discovery of mAbs against complex targets often missed by ELISA or immunoblots.

mAb 2497A remained detectable from 1 μg/mL to 1 ng/mL. The high frequency of somatic hypermutations indicates cycles of antigen-driven affinity maturation within germinal centers, consistent with antigen exposure and T-cell–dependent B-cell responses that may underlie pathogenic autoantibody production. Furthermore, patient-derived mAb 2497A recapitulated the epitope specificity and isoform selectivity observed in the corresponding serum, supporting its use as a representative probe of MOG autoantibody responses, although polyclonal humoral responses likely drive serum reactivity.

Despite these strengths, the platform has limitations. Overcalling hits by the automated algorithm and false positives (11/12, 91.6%) reflect challenges in controlling antibody concentration and nonspecific binding, as well as variability in transient MOG surface expression on HEK293T reporter cells. These findings underscore the importance of off-platform validation to avoid overestimating hits. Refining assay design and optimizing antigen expression—stable vs transient transfection—could improve specificity. The low MOG-IgG hit rate (0–0.02%) underscores the rarity of circulating antigen-specific B cells, suggesting enrichment strategies could enhance sensitivity. Furthermore, the current workflow focuses on IgG; future iterations incorporating IgA and IgM isotypes may detect clinically relevant autoantibodies, given IgA's potential diagnostic relevance in MOGAD.^15^

In summary, this optofluidic CBA-based workflow enables rapid, on-platform screening of autoreactive B cells to isolate rare autoantibodies in B-cell–mediated autoimmune diseases.
